# LLM-based cell type annotation harmonization across single-cell studies using GCTHarmony

**DOI:** 10.1186/s13059-026-04231-7

**Published:** 2026-08-04

**Authors:** Xingyuan Zhang, Zhicheng Ji

**Affiliations:** 1 Department of Biostatistics and Bioinformatics, Duke University School of Medicine, Durham, NC USA; 2 Computational Biology and Bioinformatics Program, Duke University School of Medicine, Durham, NC USA

## Abstract

A major challenge in integrating previously analyzed single-cell RNA-seq studies is the inconsistency of cell type annotations. To address this, we developed GCTHarmony, an LLM-based method for harmonizing cell type annotations across single-cell studies. Utilizing OpenAI’s text embedding model, GCTHarmony accurately maps arbitrary cell type annotations to standardized cell ontology terms and reconciles discrepancies in annotation hierarchies across studies. In real data examples, we show that GCTHarmony substantially improves the consistency of cell type annotations across single-cell studies.

**Supplementary Information:**

The online version contains supplementary material available at https://doi.org/10.1186/s13059-026-04231-7.

## Background

Single-cell RNA sequencing (scRNA-seq) technologies have evolved dramatically over the past decade. The emergence of commercial platforms such as 10x Genomics Chromium [1] has enabled the capture of tens of thousands of individual cells in a single run at low cost. In recent years, a massive number of scRNA-seq studies have been published, often investigating similar tissues or biological questions [2–5]. For example, large consortia such as HuBMAP [3], Tabula Sapiens [4], and SenNet [5] have all generated scRNA-seq data covering normal human tissues in major organs such as the liver, lung, heart, and pancreas. Integrating knowledge across these studies could greatly enhance statistical power and lead to more generalizable discoveries, transforming the fragmented patchwork of results from isolated single-cell studies into cumulative knowledge.

To effectively analyze scRNA-seq data from different studies, one must first identify a set of cell type annotations shared across studies before examining differences in cell type proportions and cell-type-specific gene expression profiles. A common approach is to integrate gene expression matrices across all studies and perform cell clustering and annotation on the integrated dataset [6, 7]. However, this method is time-consuming, requires additional manual effort, and may become computationally infeasible when large datasets are involved. An alternative is to leverage existing cell type annotations from published studies, which avoids extra manual effort and is more computationally efficient. However, substantial discrepancies in cell type annotations across studies often make them not directly comparable. These discrepancies arise primarily from two sources. First, different studies may use varying terms to describe the same cell type, such as “T-cells” versus “T cell”. While humans can easily recognize and resolve such inconsistencies, automated resolution requires complex natural language processing. Second, studies may differ in the hierarchical granularity of their annotations, with some distinguishing T cell subtypes such as naive and effector T cells, and others grouping them together. Addressing this issue requires mapping annotations to a unified cell type ontology.

Large language models (LLMs), such as GPT-4 developed by OpenAI [8], have been shown to excel in cell type annotation tasks [9–11]. When provided with a list of marker genes in the prompt message, LLMs can accurately infer corresponding cell types in scRNA-seq studies. However, directly querying LLMs via prompt messages is not an optimal strategy for mapping arbitrary cell type names to standardized cell ontology (CL) terms, for two primary reasons. First, LLMs may produce plausible but incorrect or non-standard terms that do not exist in the official ontology, a phenomenon known as AI hallucination. Second, the number of candidate ontology terms is substantial, and including all of them in a single prompt typically exceeds the context window limitations of most LLMs, making exhaustive and accurate comparison impractical. A recently published method, uHAF [12], provides a cell-type mapping agent that leverages GPT-4 to map arbitrary cell-type names to organ-specific hierarchical cell-type trees, representing another LLM-based approach to standardizing cell-type nomenclature. In contrast to prompt-based mapping to organ-specific hierarchies, we sought to develop a tissue-invariant framework that combines ontology-constrained semantic mapping with reconciliation of annotation granularity across studies.

## Results

**Fig. 1:**
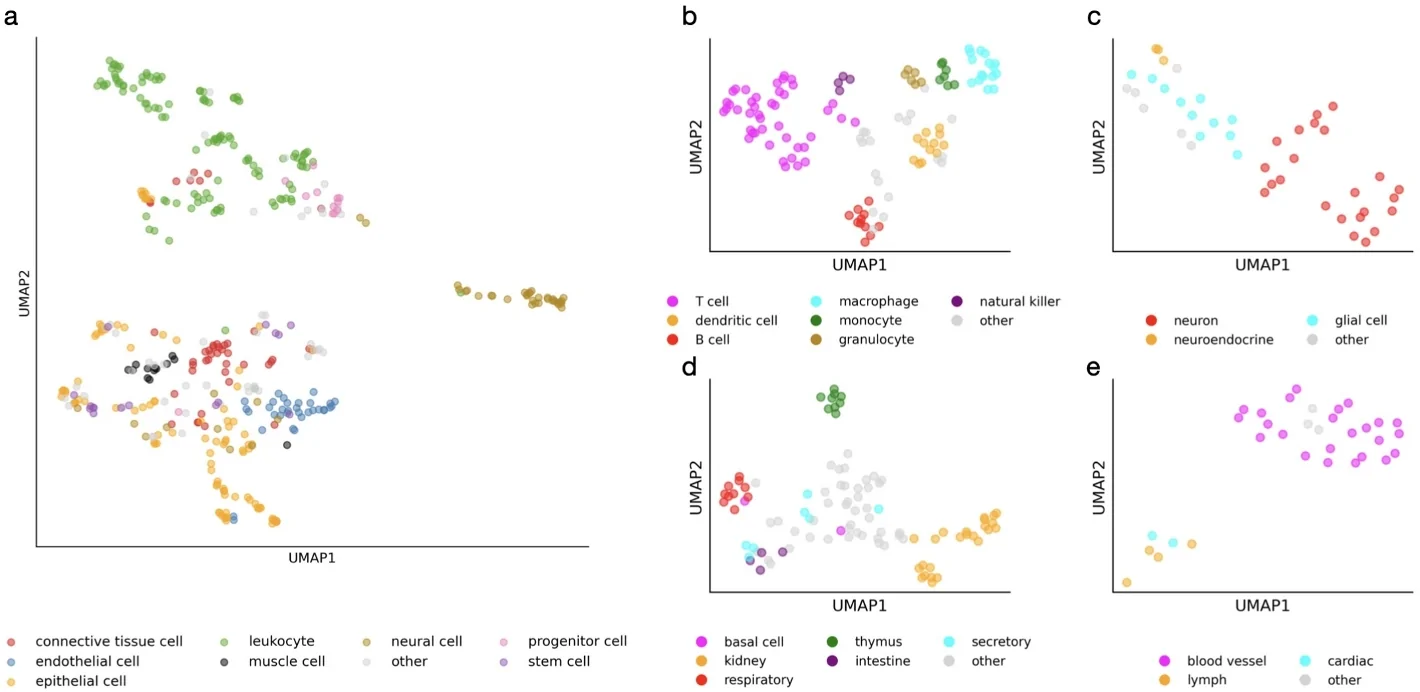
UMAP visualization of numerical embeddings by OpenAI’s text embedding model of 424 commonly referenced cell types in the Human Reference Atlas (**a**), cell types belonging to immune cells (**b**), cell types belonging to neural cells (**c**), cell types belonging to epithelial cells (**d**), and cell types belonging to endothelial cells (**e**)

Motivated by work such as GenePT [13], we explored the use of OpenAI’s text embedding model, which transforms arbitrary text inputs into numerical embedding vectors. The semantic similarity between two texts is then quantified by the distance between their corresponding vectors. To assess the suitability of this embedding model for cell type annotation, we visualized the embedding vectors of 424 commonly referenced cell types cataloged in the Human Reference Atlas (Methods) using Uniform Manifold Approximation and Projection (UMAP) for dimensionality reduction (Fig. 1a). The results show that cell type names within the same broad category cluster closely together, while those from different categories are clearly separated. Furthermore, within major categories such as leukocytes, neural cells, epithelial cells, and endothelial cells, cell type names belonging to the same sub-category also form distinct sub-clusters (Fig. 1b–e). These findings suggest that the embedding space encodes biologically meaningful relationships among cell type names and supports its potential utility for mapping arbitrary input names to standardized CL terms.

**Fig. 2:**
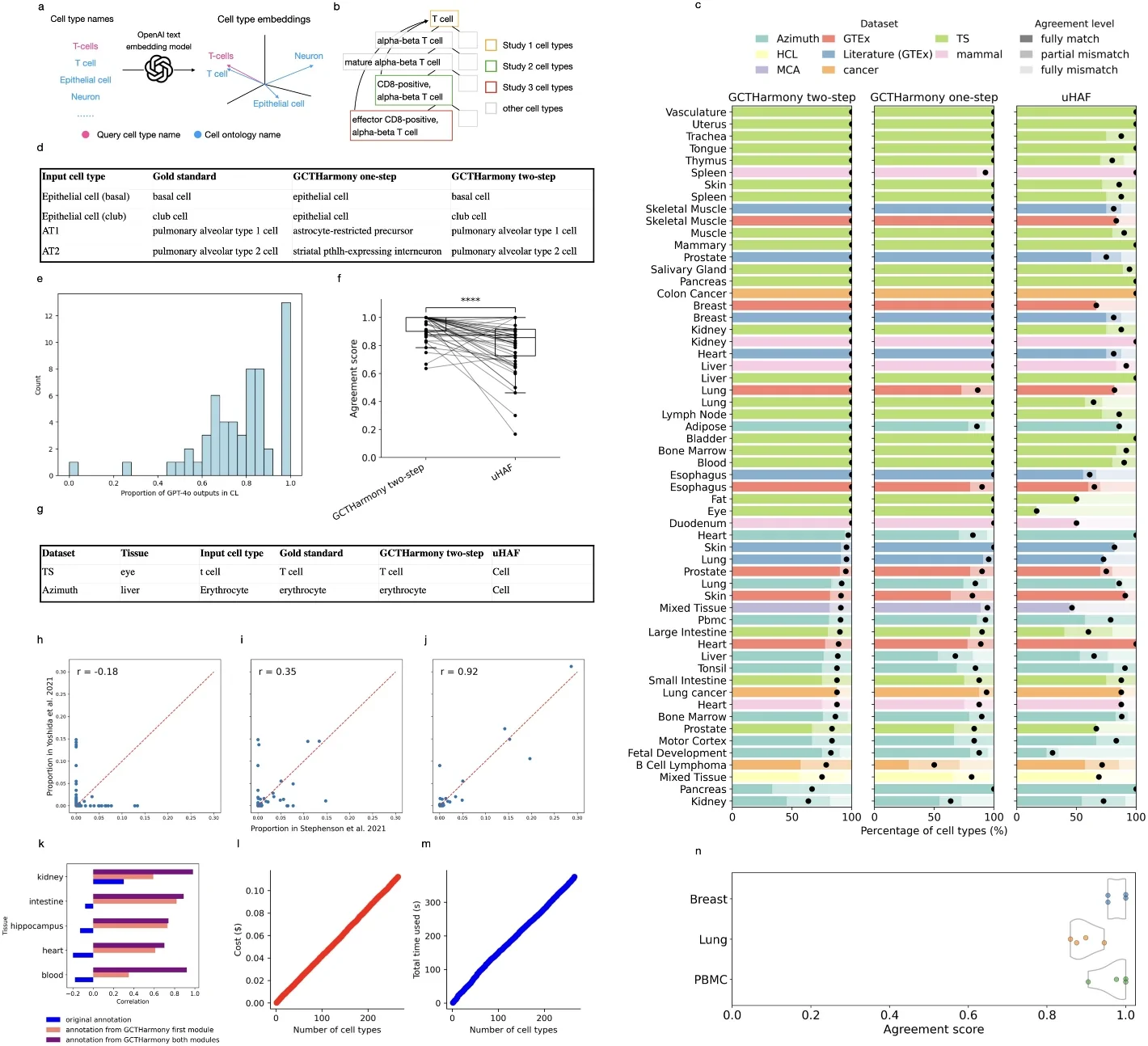
**a**, **b** Cartoons demonstrating the first (**a**) and second (**b**) modules of GCTHarmony. **c** Proportion of cell types with varying agreement levels in each study and tissue for the two strategies of GCTHarmony and uHAF. **d** Four example cell type names, their gold standard CL terms, and the CL terms assigned by the two strategies of GCTHarmony. **e** Distribution of the proportion of prompt-based mappings in which GPT-4o outputs valid CL terms across tissue–dataset pairs. **f** Agreement scores of GCTHarmony (two-step) and uHAF across tissue-dataset pairs. Statistical significance was assessed using a paired Wilcoxon signed-rank test. **** denotes $P < 1\times 10^{-4}$. **g** Two example cell type names, their gold standard CL terms, and the outputs of GCTHarmony two-step strategy and uHAF. **h**–**j** Scatter plots comparing cell type proportions in two human blood scRNA-seq studies using the original annotations (**h**), annotations from the first module of GCTHarmony (**i**), and annotations from both modules of GCTHarmony (**j**). The correlation of cell type proportions is shown in the top left corner of each plot. **k** Correlation of cell type proportions based on the original annotations, annotations from the first module of GCTHarmony, and annotations from both modules of GCTHarmony across different tissue types. **l**, **m** Financial cost (**l**) and computational time (**m**) of processing different numbers of cell types using GCTHarmony. **n** Reproducibility of GCTHarmony. Each data point represents the agreement score from a single tissue (from Azimuth) for each GCTHarmony run

Based on this finding, we developed GCTHarmony, an LLM-based method for harmonizing cell type annotations across single-cell studies (Methods). GCTHarmony has two modules. The first module maps an arbitrary cell type name to its corresponding CL term (Fig. 2a). For example, “T-cells” is mapped to the CL term “T cell” (CL:0000084). GCTHarmony utilizes OpenAI’s text embedding model to convert both the arbitrary cell type name and each CL term into numerical embeddings. Cosine similarity is then computed between the embedding of the arbitrary name and each CL term. The CL term with the highest cosine similarity is assigned to the arbitrary cell type name. After identifying CL terms in the first module, GCTHarmony resolves hierarchical discrepancies in the second module (Fig. 2b) by mapping cell subtypes (e.g., naive T cell) to their broader parent types (e.g., T cell) based on the ontology tree.

We first evaluated the accuracy of GCTHarmony’s first module, which maps cell type names to CL terms, using two distinct strategies. The first strategy (one-step) directly converts the user-provided cell type names and the commonly used CL terms into embedding vectors using the text embedding model. The second strategy (two-step) first queries the GPT-4o model to generate one-sentence descriptions for both the user-provided cell type names and the commonly used CL terms, and then converts these descriptions into embedding vectors using the text embedding model. Using benchmark datasets and following the evaluation procedure of our previous study [9], we compared the CL terms generated by both strategies to manually annotated CL terms, treated as the gold standard, across a variety of tissue types and datasets (Fig. 2c). Only gold standard CL terms appearing in a curated list of commonly used terms from the Human Reference Atlas (HRA) were included in the evaluation, since these represent the cell type annotations most frequently encountered in real practice. The CL terms generated by both strategies show strong concordance with the gold-standard annotations. The two-step strategy achieves a mean agreement score of 0.945 and a median agreement score of 1 across tissue–dataset pairs. 70% of pairs exceed 90% full agreement, and 96% exceed 90% when full and partial agreement are combined. The one-step strategy achieves a mean agreement score of 0.934 and a median agreement score of 1 across tissue–dataset pairs. 61% of pairs exceed 90% full agreement, and 95% exceed 90% when full and partial agreement are combined. The error rates, defined as the proportion of fully mismatched annotations, average 1% for the two-step strategy and 2% for the one-step strategy across all tissue–dataset pairs. The improved performance of the two-step strategy is likely due to the additional semantic enrichment provided by GPT-4o, which allows the text embedding model to better capture the intended meaning of abbreviated or ambiguous terms. Figure 2d presents example cell type names where the one-step strategy failed but the two-step strategy produced correct matches. These examples involve semantically complex names (e.g., those with parentheses) or abbreviations (e.g., “AT1”), which cannot be effectively handled by the embedding model alone. By default, GCTHarmony uses the two-step strategy due to its superior performance, although the one-step strategy is also provided as a simpler alternative.

To demonstrate the advantage of GCTHarmony’s embedding-based approach, we evaluated an alternative prompt-based strategy that directly queried GPT-4o for the CL term corresponding to each custom cell-type annotation. Even with strict prompting constraints requiring the model to return only valid CL names, the LLM frequently generated outputs that do not belong to CL (Fig. 2e). In 74% of tissue–dataset pairs, fewer than 90% of the outputs correspond to valid CL terms. By contrast, GCTHarmony’s design guarantees that all predicted labels belong to valid CL terms. These results highlight the limited reliability of direct LLM-based mapping and underscore the importance of incorporating a text-embedding model to ensure ontology-valid predictions. Given this poor performance, the prompt-based strategy is not considered further in subsequent analyses.

We further compared GCTHarmony with uHAF [12] (Fig. 2c, Methods). Although both methods aim to standardize cell-type annotations, GCTHarmony differs fundamentally by relying on an embedding-based approach with a tissue-invariant ontology, rather than on LLM-prompt interactions and tissue-specific ontologies. uHAF achieves a mean agreement score of 0.811 and a median agreement score of 0.857 across tissue–dataset pairs, significantly lower than the performance of GCTHarmony (Fig. 2f). A major contributing factor is that uHAF-T is not comprehensive and omits many cell types present in real datasets. For instance, T cells, which play an essential role in the immune system of the eye [14], are absent from uHAF-T (Fig. 2g). Similarly, the liver is an important site for the erythrocyte life cycle [15], but erythrocytes in the liver are absent from uHAF-T (Fig. 2g).

In addition to higher accuracy, GCTHarmony offers several practical advantages over uHAF. uHAF depends on an online interface and substantial manual operations, which limits scalability and makes it impractical for large collections of datasets. GCTHarmony is fully automated and requires no manual intervention beyond standard programming, making it well suited for large-scale analyses. Because uHAF relies on tissue-specific ontologies and requires users to specify a tissue type, it cannot support cross-tissue or mixed-tissue harmonization, whereas GCTHarmony uses a tissue-invariant ontology that naturally enables such analyses. Finally, GCTHarmony can harmonize any user-defined labels through a general embedding function, enabling the use of custom ontologies and extending GCTHarmony beyond cell-type annotations, whereas uHAF is restricted to cell-type annotations and a fixed ontology.

We next evaluated the full GCTHarmony method for harmonizing cell type annotations across different single-cell studies. We collected five pairs of scRNA-seq datasets (Methods), with each pair derived from the same tissue type (e.g., blood) but generated and analyzed by different research groups. Since each pair was derived from the same tissue, their cell type compositions are expected to be consistent. We compared the correlation of cell type proportions between the two studies in each pair using the annotations downloaded from the original studies, CL terms generated by GCTHarmony’s first module, and CL terms produced by the full GCTHarmony pipeline. For cell types present in only one study, we assigned a proportion of zero in the other study. Figure 2h–j show example correlations for the blood tissue, and Fig. 2k summarizes the correlations across all five dataset pairs. Using the original annotations, the correlations were often negative, primarily due to inconsistent naming. This inconsistency was largely resolved after mapping cell type names to CL terms using GCTHarmony’s first module, which substantially improved the correlations. The second module further enhanced consistency by addressing hierarchical discrepancies, resulting in the highest overall correlations. These results suggest that GCTHarmony can robustly harmonize cell type annotations across diverse tissue types.

Similar to GPTCelltype [9], the first module of GCTHarmony involves the use of the OpenAI API, which is not free of charge. However, the cost is often negligible in real applications. For example, processing 100 cell types costs only around 4 US cents (Fig. 2l) for full GCTHarmony method. GCTHarmony is also computationally efficient, requiring only about 100 seconds to process 100 cell types (Fig. 2m). Its results are highly reproducible, with agreement scores showing strong consistency across individual runs (Fig. 2n). These results suggest that GCTHarmony is a cost-effective and reproducible approach in practice.

Since OpenAI models are not universally accessible, we also evaluated the performance of another widely used model, Qwen3-Embedding-0.6B, which has fewer parameters than models such as GPT-4o. Qwen3 produces an embedding space that cleanly separates major cell types and subtypes (Additional file: Fig. S1), although its mapping accuracy is lower than that of OpenAI’s text-embedding model (Additional file: Fig. S2). Using Qwen3, the one-step approach yields a mean agreement score of 0.912 and a median of 1, whereas the two-step approach achieves a mean of 0.836 and a median of 0.875.

The commonly used CL terms compiled from HRA, which serve as the default in GCTHarmony, cover a median of 80% of the gold standard CL terms across tissue–dataset pairs in the benchmark dataset [9]. GCTHarmony also allows users to employ the full set of CL terms, although this choice results in a slightly lower agreement score and increased computational time (Additional file: Fig. S3).

We also explored incorporating additional contextual information from the title and abstract of the original publication into the prompt, beyond using the cell type name alone. However, this did not improve performance in the two-step strategy and substantially decreased performance in the one-step strategy (Additional file: Fig. S4). A potential explanation is that titles and abstracts often contain broad biological context that is not directly relevant to the specific cell types being harmonized, which may introduce noise or divert the model’s attention from the core cell identity.

## Discussion

GCTHarmony advances LLM-based label harmonization by reframing the task as a cross-study annotation integration problem that requires both semantic standardization and reconciliation of annotation granularity. Its contribution is primarily conceptual and practical, providing a unified framework that converts heterogeneous free-text labels into standardized and comparable ontology terms across independently analyzed studies. To our knowledge, text-embedding-based, closed-vocabulary retrieval has not been systematically developed and evaluated for harmonizing free-text cell-type annotations across single-cell studies. This design also supports the direct reuse of existing annotations without reprocessing the underlying gene-expression data.

The framework also provides conceptual and methodological distinctions from previous prompt-based approaches. Although uHAF similarly addresses hierarchical cell-type standardization, it uses LLM prompting with organ-specific hierarchical trees. GCTHarmony instead maps annotations to the broadly applicable Cell Ontology and then reconciles the resulting terms across studies, enabling cross-tissue and mixed-tissue analyses without requiring a predefined organ-specific hierarchy. Standard text embeddings and similarity search provide a simple and scalable semantic retrieval mechanism, while the broader methodological contribution lies in integrating this mechanism into an ontology-constrained framework that ensures valid outputs, broad ontology coverage, tissue-independent application, and explicit reconciliation of annotation depth.

## Conclusions

In summary, we developed GCTHarmony, an LLM-based method that accurately maps cell type names to CL terms and harmonizes cell type annotations across single-cell studies. We showed that GCTHarmony improves the consistency of cell type proportions in real data examples. GCTHarmony will streamline the integration of multiple single-cell studies processed and analyzed by different research groups. GCTHarmony further highlights the strengths of an embedding-based approach, suggesting a promising direction for future methods aimed at standardizing cell type annotations.

## Methods

### Obtaining Cell Ontology (CL) terms

Cell Ontology (CL) terms were downloaded from the Open Biological and Biomedical Ontologies (OBO) Foundry on January 25, 2025 (http://purl.obolibrary.org/obo/cl.owl). The Python package owlready2 [16] was used to manipulate the Web Ontology Language (OWL) ontologies. A total of 3076 CL terms were obtained.

Commonly used CL terms were obtained from the ASCT+B Tables available through the Human Reference Atlas (HRA) portal (https://humanatlas.io/asctb-tables) [17]. In each table, CL terms are listed under the column “CT/1/LABEL”. By concatenating all CL terms across anatomical structures and removing duplicates, a total of 424 unique CL terms were obtained.

### Visualizing CL terms

Each commonly used CL term was converted into an embedding vector using OpenAI’s text-embedding-3-large model or Qwen3-Embedding-0.6B model. We manually annotated the broad cell type category for each CL term. The embedding vectors of all commonly used CL terms, or of CL terms belonging to a specific broad category, were concatenated into an embedding matrix. The Python package umap [18] was applied to the embedding matrix using default settings, and the resulting UMAP dimensions were subsequently visualized in Fig. 1 and in Additional file: Fig. S1.

### GCTHarmony

#### First module: mapping cell type names to CL terms

GCTHarmony provides two strategies for mapping cell type names to CL terms. In the one-step strategy, the user-provided cell type name is directly converted into a numerical embedding vector using OpenAI’s text-embedding-3-large model. In the two-step strategy, the gpt-4o-2024-08-06 model is first queried via the OpenAI API with the prompt: “Please use 1 sentence to describe cell type: INPUT-CELL-TYPE”, where INPUT-CELL-TYPE is the user-provided cell type name. The response from GPT-4o is then converted into a numerical embedding vector using the text-embedding-3-large model. The same procedure was used to obtain numerical embeddings of all CL terms in both strategies. The two-step strategy is used by default.

Denote $\boldsymbol{z}$ as the embedding of the user-provided cell type name and $\boldsymbol{v}_i$ as the embedding of the *i*th CL term. The user-provided cell type name is then mapped to the *j*th CL term with the highest cosine similarity. Specifically:

$$\begin{aligned} j = \arg \max _{\boldsymbol{v}_i \in \mathcal {V}} \, \frac{\boldsymbol{z} \cdot \boldsymbol{v}_i}{\Vert \boldsymbol{z}\Vert \, \Vert \boldsymbol{v}_i\Vert } \end{aligned}$$

Here, $\mathcal {V}$ is the set of all CL terms being considered. By default, $\mathcal {V}$ includes only the commonly used CL terms, as many CL terms are rarely used. Users also have the option to set $\mathcal {V}$ to include all CL terms.

#### Second module: reconciling hierarchical discrepancies

Let there be K studies. After applying the first module of GCTHarmony to each study k, we obtain a set of CL terms:

$$\begin{aligned} S_k = \{ c_{k1}, c_{k2}, \ldots \}. \end{aligned}$$

GCTHarmony constructs the union of all predicted CL terms across studies:

$$\begin{aligned} U = \bigcup _{k=1}^{K} S_k. \end{aligned}$$

Let the Cell Ontology be represented as a rooted tree T with root r. For any node v in the ontology tree, let

$$\begin{aligned} \textrm{Desc}(v) = \{ u \in T \mid u \text { is a descendant of } v \}. \end{aligned}$$

GCTHarmony then performs a top down traversal of the ontology tree starting from r. For every node v that appears in the union set,

$$\begin{aligned} v \in U, \end{aligned}$$

GCTHarmony assigns the name of v to all of its descendant terms that also belong to the union set:

$$\begin{aligned} \forall u \in \left( U \cap \textrm{Desc}(v) \right) , \quad \text {assign } u \leftarrow v. \end{aligned}$$

In other words, each term in the union set inherits the name of the highest level ancestor in U during the traversal.

Using Fig. 1b as an example, consider a scenario where one study contains a cell type harmonized to “T cell”, while two other studies assign more specific labels such as “CD8-positive, alpha-beta T cell” and “effector CD8-positive, alpha-beta T cell”. Because both specific subtypes are descendants of the broader term “T cell”, the module reconciles these discrepancies by collapsing all three labels to their shared ancestral node “T cell”.

#### Qwen3-0.6B model

To support users without access to OpenAI models, we provide an alternative built-in backend based on the Qwen3-0.6B model, enabling full use of GCTHarmony without external API dependencies. By default, if an OpenAI API key is not provided, GCTHarmony automatically switches to the Qwen3-0.6B backend.

The overall annotation strategy using Qwen3-0.6B mirrors that of the GPT-based pipeline. In the one-step strategy, GCTHarmony directly generates embedding vectors for each input cell type using the Qwen3-Embedding-0.6B model. In the two-step strategy, GCTHarmony first queries the Qwen3-0.6B language model to generate a description using the same prompt: “Please use 1 sentence to describe cell type: INPUT-CELL-TYPE”. The resulting description is then embedded using the Qwen3-Embedding-0.6B model.

#### Context-augmented annotation strategy

We implemented a context-augmented annotation strategy within the GCTHarmony framework, allowing users to include contextual information directly in the input message. In the one-step strategy, the cell type name and its contextual information are concatenated into a single input string and embedded using the selected embedding model. In the two-step strategy, GCTHarmony first queries the language model to generate a concise, single-sentence description of the cell type name combined with the contextual information, and then embeds the resulting description to obtain the final embedding.

### Competing methods

#### Prompt-based mapping with GPT-4o

The gpt-4o-2024-08-06 model was queried directly using the prompt, “What is the cell ontology label for ‘INPUT-CELL-TYPE’? Only respond with the exact ontology label, and nothing else. Do not include definitions, IDs, or any explanation. Output only the ontology name.” The model’s response was taken as the harmonized CL term.

#### uHAF

The default uHAF pipeline was applied to the same benchmark dataset used for GCTHarmony. For each dataset–tissue pair, custom cell-type names and tissue information were provided as input to the uHAF agent. For tissues not documented in uHAF-T, the closest available tissue was selected. After obtaining the harmonized uHAF-T nodes for each cell type, these nodes were manually mapped to the closest standard CL terms. The evaluation procedure was then performed using the same protocol as for GCTHarmony.

### Collection of paired scRNA-seq datasets for benchmarking

Five pairs of scRNA-seq datasets used for benchmarking were all downloaded from publicly available sources. The two blood datasets were obtained from [19, 20]. The two heart datasets were obtained from [21, 22]. The two hippocampus datasets were obtained from [23, 24]. The two intestine datasets were obtained from [25, 26]. The two kidney datasets were obtained from [27, 28].

Cell type annotations provided by the original studies were directly obtained from their published data.

### Financial cost

The total financial cost of using GCTHarmony is calculated based on the API pricing of OpenAI’s text-embedding-3-large model and the gpt-4o-2024-08-06 model. Specifically, the cost of the text-embedding-3-large model is $0.00013 per 1,000 tokens. The cost of gpt-4o-2024-08-06 model is $0.0025 per 1,000 input tokens and $0.01 per 1,000 output tokens.

### Computational time

To evaluate the computational efficiency of GCTHarmony, we benchmarked its runtime on a machine with an Intel(R) Xeon(R) CPU E5-2699 v4 @ 2.20GHz processor and 64 GB of RAM.

### Reproducibility analysis

To benchmark the reproducibility of GCTHarmony, we applied it to three example tissue types, repeating the process four times. For each tissue, an agreement score was calculated between the gold standard and each GCTHarmony run.

## Supplementary information

Additional file 1. Supplementary figures. Figure S1. UMAP visualization of numerical embeddings generated by the Qwen3-Embedding-0.6B model for 424 commonly referenced cell types in the Human Reference Atlas and in selected major cell-type categories. Figure S2. Proportion of cell types with varying agreement levels across studies and tissues for the two-step strategy and one-step strategy using Qwen3. Figure S3. Median agreement score and runtime comparing the use of commonly used CL terms with all CL terms in GCTHarmony two-step strategy. Figure S4. Proportion of cell types with different agreement levels across studies and tissues for the two-step and one-step strategies of GCTHarmony augmented by publication titles and abstracts.

## Data Availability

The five pairs of scRNA-seq datasets used for benchmarking were obtained from publicly available repositories. The two blood datasets were obtained from Stephenson et al. [19], deposited in ArrayExpress under accession E-MTAB-10026 [29], and Yoshida et al. [20], deposited in the European Genome–Phenome Archive under accession EGAD00001007718 [30]; the two heart datasets were obtained from Tucker et al. [21], deposited in the Broad Institute Single Cell Portal under study accession SCP498 [31], and Litviňuková et al. [22], deposited in the European Nucleotide Archive under accession ERP123138 [32]; the two hippocampus datasets were obtained from Ayhan et al. [23], deposited in the Gene Expression Omnibus under accession GSE160189 [33], and Franjic et al. [24], deposited in the Gene Expression Omnibus under accession GSE186538 [34]; the two intestine datasets were obtained from Smillie et al. [25], deposited in the Broad Institute Single Cell Portal under study accession SCP259 [35], and Elmentaite et al. [26], and are associated with ArrayExpress accession E-MTAB-9543 [36].; and the two kidney datasets were obtained from Lake et al. [27], deposited in the Gene Expression Omnibus under accession GSE183279 [37], and from the Human Cell Atlas Kidney Seed Network, deposited in the Human Cell Atlas Data Explorer under project identifier 29ed827b- c539-4f4c-bb6b-ce8f9173dfb7 [38]. Cell type annotations were obtained directly from the datasets released by the original studies. The GCTHarmony package is open-source Python software available on GitHub [39] at https://github.com/Xingyuan-Zhang/GCTHarmony and on Zenodo [40] at https://zenodo.org/records/21513453. Both versions are distributed under the MIT License. The benchmarking datasets and analysis scripts are available on GitHub [41] at https://github.com/Xingyuan-Zhang/GCTHarmony_analysis and on Zenodo [42] at https://doi.org/10.5281/zenodo.21519264, also under the MIT License.

## References

[1] Zheng GX, Terry JM, Belgrader P, Ryvkin P, Bent ZW, Wilson R, et al. Massively parallel digital transcriptional profiling of single cells. Nat Commun. 2017;8(1):14049. 10.1038/ncomms14049 PMC5241818 28091601

[2] Regev A, Teichmann SA, Lander ES, Amit I, Benoist C, Birney E, et al. The human cell atlas. Elife. 2017;6:e27041. 10.7554/eLife.27041 PMC5762154 29206104

[3] Consortium H. The human body at cellular resolution: the NIH Human Biomolecular Atlas Program. Nature. 2019;574(7777):187–92. 10.1038/s41586-019-1629-x PMC6800388 31597973

[4] Consortium TTS, Jones RC, Karkanias J, Krasnow MA, Pisco AO, Quake SR, et al. The Tabula Sapiens: A multiple-organ, single-cell transcriptomic atlas of humans. Science. 2022;376(6594):eabl4896. 10.1126/science.abl4896 PMC9812260 35549404

[5] Consortium S. NIH SenNet Consortium to map senescent cells throughout the human lifespan to understand physiological health. Nat Aging. 2022;2(12):1090–100. 10.1038/s43587-022-00326-5 PMC10019484 36936385

[6] Stuart T, Butler A, Hoffman P, Hafemeister C, Papalexi E, Mauck WM. Comprehensive integration of single-cell data. Cell. 2019;177(7):1888–902. 10.1016/j.cell.2019.05.031 PMC6687398 31178118

[7] Korsunsky I, Millard N, Fan J, Slowikowski K, Zhang F, Wei K, et al. Fast, sensitive and accurate integration of single-cell data with Harmony. Nat Methods. 2019;16(12):1289–96. 10.1038/s41592-019-0619-0 PMC6884693 31740819

[8] Achiam J, Adler S, Agarwal S, Ahmad L, Akkaya I, Aleman FL. Gpt-4 technical report. 2023. arXiv preprint arXiv:2303.08774.

[9] Hou W, Ji Z. Assessing GPT-4 for cell type annotation in single-cell RNA-seq analysis. Nat Methods. 2024;21(8):1462–5. 10.1038/s41592-024-02235-4 PMC11310073 38528186

[10] Chen J, Zhang J, Yao H, Li Y. Celltypeagent: Trustworthy cell type annotation with large language models. 2025. arXiv preprint arXiv:2505.08844.

[11] Crowley G, Consortium TS, Quake SR. Benchmarking cell type and gene set annotation by large language models with AnnDictionary. Nat Commun. 2025;16:9511. 10.1038/s41467-025-64511-x PMC12569162 41152246

[12] Bian H, Chen Y, Wei L, Zhang X. UHAF: a unified hierarchical annotation framework for cell type standardization and harmonization. Bioinformatics. 2025;41(4):btaf149. 10.1093/bioinformatics/btaf149 PMC12002906 40172934

[13] Chen Y, Zou J. Simple and effective embedding model for single-cell biology built from ChatGPT. Nat Biomed Eng. 2025;9(4):483–93. 10.1038/s41551-024-01284-6 39643729

[14] Loi JK, Alexandre YO, Senthil K, Schienstock D, Sandford S, Devi S. Corneal tissue-resident memory T cells form a unique immune compartment at the ocular surface. Cell Rep. 2022. 10.1016/j.celrep.2022.110852. 35613584

[15] Theurl I, Hilgendorf I, Nairz M, Tymoszuk P, Haschka D, Asshoff M, et al. On-demand erythrocyte disposal and iron recycling requires transient macrophages in the liver. Nat Med. 2016;22(8):945–51. 10.1038/nm.4146 PMC4957133 27428900

[16] Lamy JB. Owlready: Ontology-oriented programming in Python with automatic classification and high level constructs for biomedical ontologies. Artif Intell Med. 2017;80:11–28. 10.1016/j.artmed.2017.07.002 28818520

[17] Börner K, Teichmann SA, Quardokus EM, Gee JC, Browne K, Osumi-Sutherland D, et al. Anatomical structures, cell types and biomarkers of the Human Reference Atlas. Nat Cell Biol. 2021;23(11):1117–28. 10.1038/s41556-021-00788-6 PMC10079270 34750582

[18] McInnes L, Healy J, Melville J. Umap: Uniform manifold approximation and projection for dimension reduction. 2018. arXiv preprint arXiv:1802.03426.

[19] Stephenson E, Reynolds G, Botting RA, Calero-Nieto FJ, Morgan MD, Tuong ZK, et al. Single-cell multi-omics analysis of the immune response in COVID-19. Nat Med. 2021;27(5):904–16. 10.1038/s41591-021-01329-2 PMC8121667 33879890

[20] Yoshida M, Worlock KB, Huang N, Lindeboom RG, Butler CR, Kumasaka N, et al. Local and systemic responses to SARS-CoV-2 infection in children and adults. Nature. 2022;602(7896):321–7. 10.1038/s41586-021-04345-x PMC8828466 34937051

[21] Tucker NR, Chaffin M, Fleming SJ, Hall AW, Parsons VA, Bedi Jr KC, et al. Transcriptional and cellular diversity of the human heart. Circulation. 2020;142(5):466–82. 10.1161/CIRCULATIONAHA.119.045401 PMC7666104 32403949

[22] Litviňuková M, Talavera-López C, Maatz H, Reichart D, Worth CL, Lindberg EL, et al. Cells of the adult human heart. Nature. 2020;588(7838):466–72. 10.1038/s41586-020-2797-4 PMC7681775 32971526

[23] Ayhan F, Kulkarni A, Berto S, Sivaprakasam K, Douglas C, Lega BC, et al. Resolving cellular and molecular diversity along the hippocampal anterior-to-posterior axis in humans. Neuron. 2021;109(13):2091–105. 10.1016/j.neuron.2021.05.003 PMC8273123 34051145

[24] Franjic D, Skarica M, Ma S, Arellano JI, Tebbenkamp AT, Choi J, et al. Transcriptomic taxonomy and neurogenic trajectories of adult human, macaque, and pig hippocampal and entorhinal cells. Neuron. 2022;110(3):452–69. 10.1016/j.neuron.2021.10.036 PMC8813897 34798047

[25] Smillie CS, Biton M, Ordovas-Montanes J, Sullivan KM, Burgin G, Graham DB, et al. Intra-and inter-cellular rewiring of the human colon during ulcerative colitis. Cell. 2019;178(3):714–30. 10.1016/j.cell.2019.06.029 PMC6662628 31348891

[26] Elmentaite R, Kumasaka N, Roberts K, Fleming A, Dann E, King HW, et al. Cells of the human intestinal tract mapped across space and time. Nature. 2021;597(7875):250–5. 10.1038/s41586-021-03852-1 PMC8426186 34497389

[27] Lake BB, Menon R, Winfree S, Hu Q, Melo Ferreira R, Kalhor K, et al. An atlas of healthy and injured cell states and niches in the human kidney. Nature. 2023;619(7970):585–94. 10.1038/s41586-023-05769-3 PMC10356613 37468583

[28] Bitzer M, Menon R, Kretzler M, Otto E, O’Connor C. HCA seed network precise tumor-nephrectomy samples. 2022. https://explore.data.humancellatlas.org/projects/29ed827b-c539-4f4c-bb6b-ce8f9173dfb7. Single-cell RNA sequencing data from normal regions of tumor-nephrectomy samples. Accessed March 29, 2025.

[29] Stephenson E, Reynolds G, Botting RA, et al. Single-cell multi-omics analysis of the immune response in COVID-19. Single-cell dataset. ArrayExpress; 2021. https://www.ebi.ac.uk/biostudies/arrayexpress/studies/E-MTAB-10026. Accession E-MTAB-10026. Accessed March 29, 2025.

[30] Yoshida M, Worlock KB, Huang N, et al. Local and systemic responses to SARS-CoV-2 infection in children and adults. Single-cell dataset. European Genome–Phenome Archive; 2022. https://ega-archive.org/datasets/EGAD00001007718. Accession EGAD00001007718. Accessed March 29, 2025.

[31] Tucker NR, Chaffin M, Fleming SJ, et al. Transcriptional and Cellular Diversity of the Human Heart. Single-cell dataset. Broad Institute Single Cell Portal; 2020. https://singlecell.broadinstitute.org/single_cell/study/SCP498. Study accession SCP498. Accessed March 29, 2025.

[32] Litviňuková M, Talavera-López C, Maatz H, et al. Cells of the adult human heart. Single-cell dataset. European Nucleotide Archive; 2020. https://www.ebi.ac.uk/ena/browser/view/ERP123138. Accession ERP123138. Accessed March 29, 2025.

[33] Ayhan F, Kulkarni A, Berto S, et al. Resolving cellular and molecular diversity along the hippocampal anterior-to-posterior axis in humans. Single-cell dataset. Gene Expression Omnibus; 2021. https://www.ncbi.nlm.nih.gov/geo/query/acc.cgi?acc=GSE160189. Accession GSE160189. Accessed March 29, 2025. 10.1016/j.neuron.2021.05.003 PMC8273123 34051145

[34] Franjic D, Skarica M, Ma S, et al. Transcriptomic taxonomy and neurogenic trajectories of adult human, macaque, and pig hippocampal and entorhinal cells. Single-cell dataset. Gene Expression Omnibus; 2022. https://www.ncbi.nlm.nih.gov/geo/query/acc.cgi?acc=GSE186538. Accession GSE186538. Accessed March 29, 2025. 10.1016/j.neuron.2021.10.036 PMC8813897 34798047

[35] Smillie CS, Biton M, Ordovas-Montanes J, et al. Intra- and Inter-cellular Rewiring of the Human Colon during Ulcerative Colitis. Single-cell dataset. Broad Institute Single Cell Portal; 2019. https://singlecell.broadinstitute.org/single_cell/study/SCP259. Study accession SCP259. Accessed March 29, 2025. 10.1016/j.cell.2019.06.029 PMC6662628 31348891

[36] Elmentaite R, Kumasaka N, Roberts K, Fleming A, Dann E, et al. The human gastrointestinal tract through space and time: Adult GEX. Single-cell dataset. ArrayExpress; 2021. https://www.ebi.ac.uk/biostudies/arrayexpress/studies/E-MTAB-9543. Accession E-MTAB-9543. Accessed March 29, 2025.

[37] Lake BB, Menon R, Winfree S, Hu Q, Melo Ferreira R, Kalhor K, et al. An atlas of healthy and injured cell states and niches in the human kidney. Single-cell dataset. Gene Expression Omnibus; 2022. https://www.ncbi.nlm.nih.gov/geo/query/acc.cgi?acc=GSE183279. GEO SuperSeries accession GSE183279. Accessed March 29, 2025.

[38] Kretzler M, Otto E, O’Connor C, Bitzer M, Menon R. HCA seed network precise tumor-nephrectomy samples. Single-cell dataset. Human Cell Atlas Data Explorer; 2022. https://explore.data.humancellatlas.org/projects/29ed827b-c539-4f4c-bb6b-ce8f9173dfb7. Project identifier 29ed827b-c539-4f4c-bb6b-ce8f9173dfb7. Accessed March 29, 2025.

[39] Zhang X, Ji Z. Software package for GCTHarmony. GitHub; 2026. https://github.com/Xingyuan-Zhang/GCTHarmony.

[40] Zhang X, Ji Z. Software package for GCTHarmony. Zenodo; 2026. 10.5281/zenodo.21513453.

[41] Zhang X, Ji Z. Analysis code for GCTHarmony. GitHub; 2026. https://github.com/Xingyuan-Zhang/GCTHarmony_analysis.

[42] Zhang X, Ji Z. Analysis code for GCTHarmony. Zenodo. 2026. 10.5281/zenodo.21519264.

