# Supplementary material for: LLM-based cell type annotation harmonization across single-cell studies using GCTHarmony
Source: Genome Biol. 2026 Aug 4;27:335. doi: 10.1186/s13059-026-04231-7 (PMC13721584; doi:10.1186/s13059-026-04231-7)
Supplement: Supplementary file 1 [file 13059_2026_4231_MOESM1_ESM.pdf]

Additional file

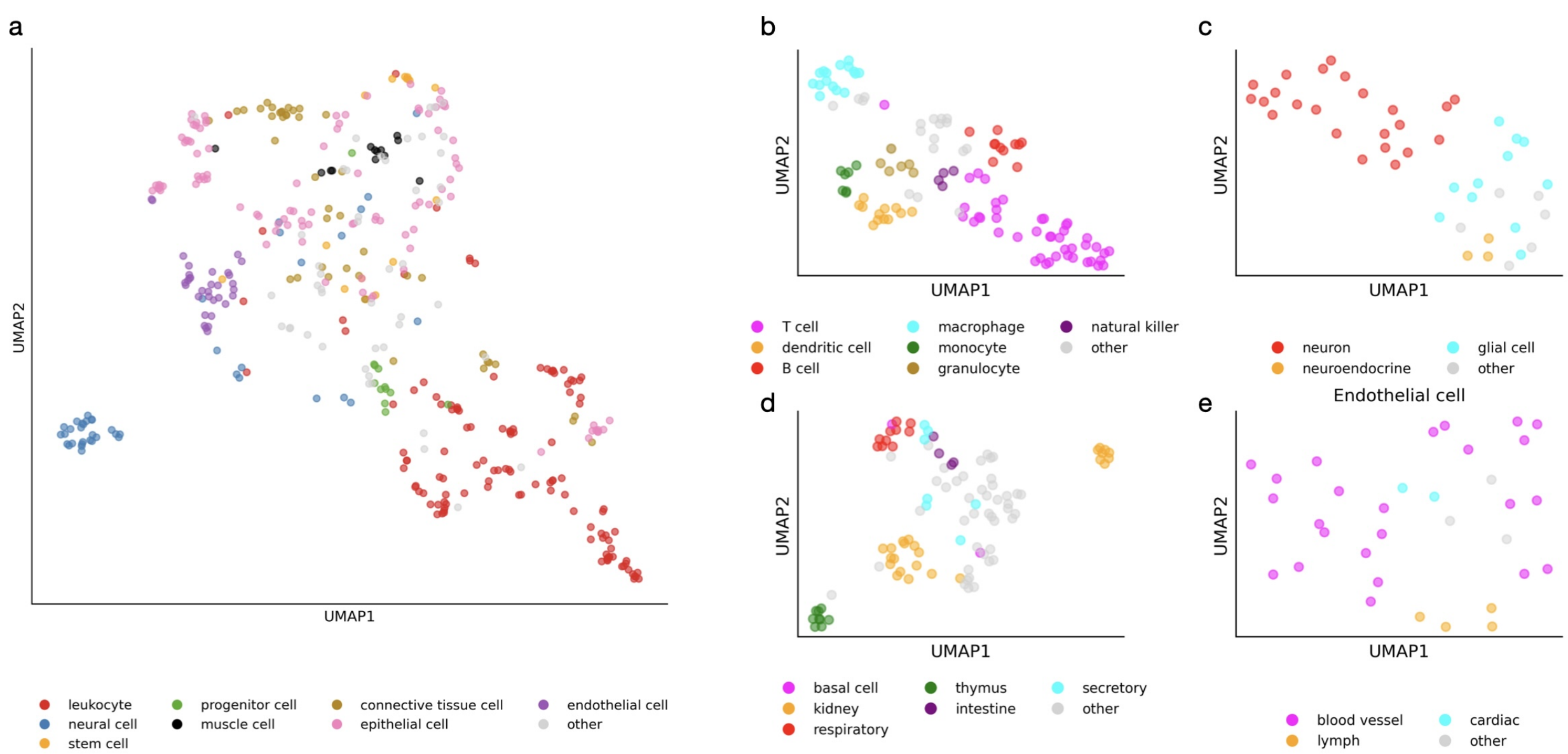

Figure S1. UMAP visualization of numerical embeddings by Qwen3-Embedding-0.6B model of 424 commonly referenced cell types in the Human Reference Atlas (**a**), cell types belonging to immune cells (**b**), cell types belonging to neural cells (**c**), cell types belonging to epithelial cells (**d**), and cell types belonging to endothelial cells (**e**).

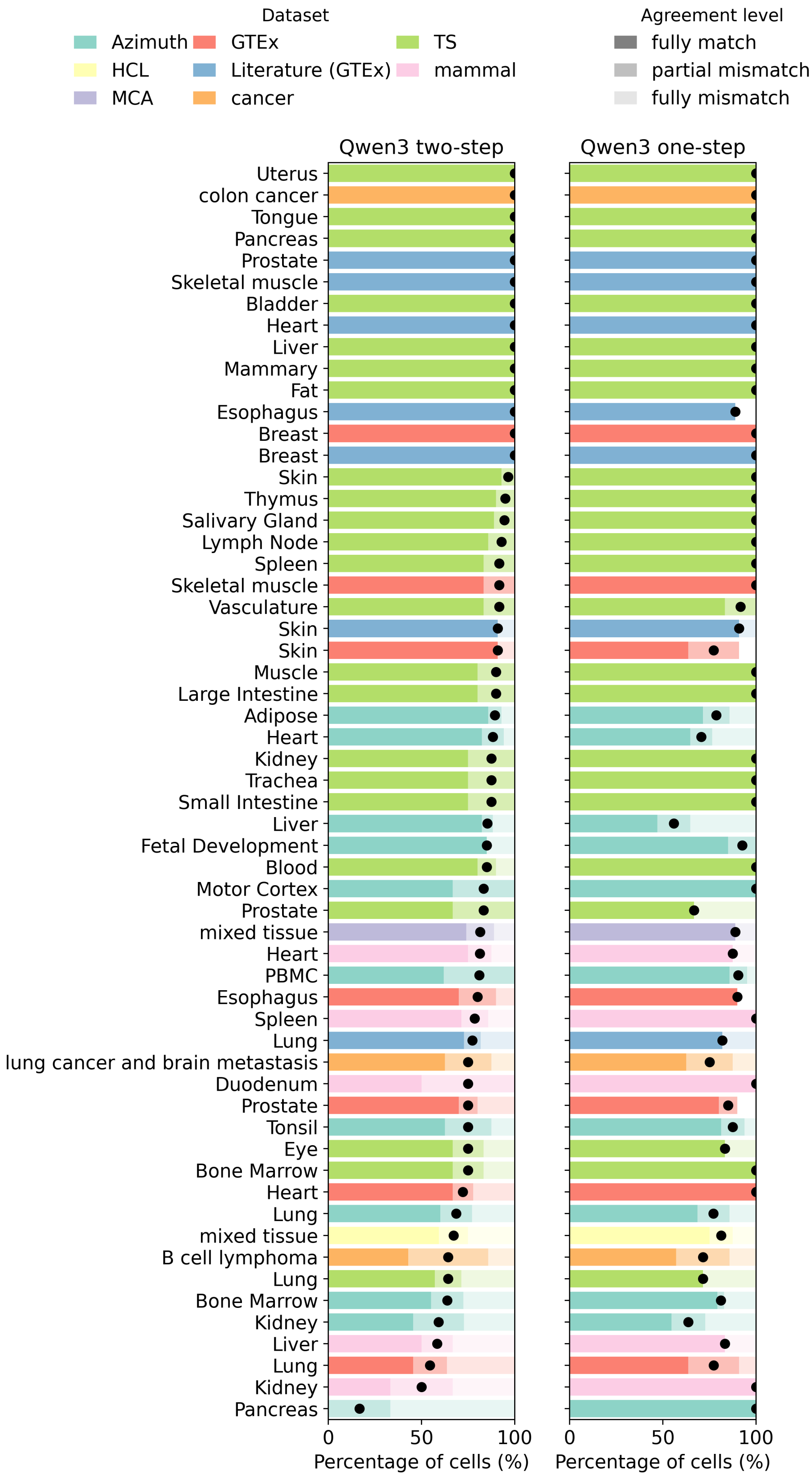

Figure S2. Proportion of cell types with varying agreement levels across studies and tissues for the two-step strategy and one-step strategy using Qwen3.

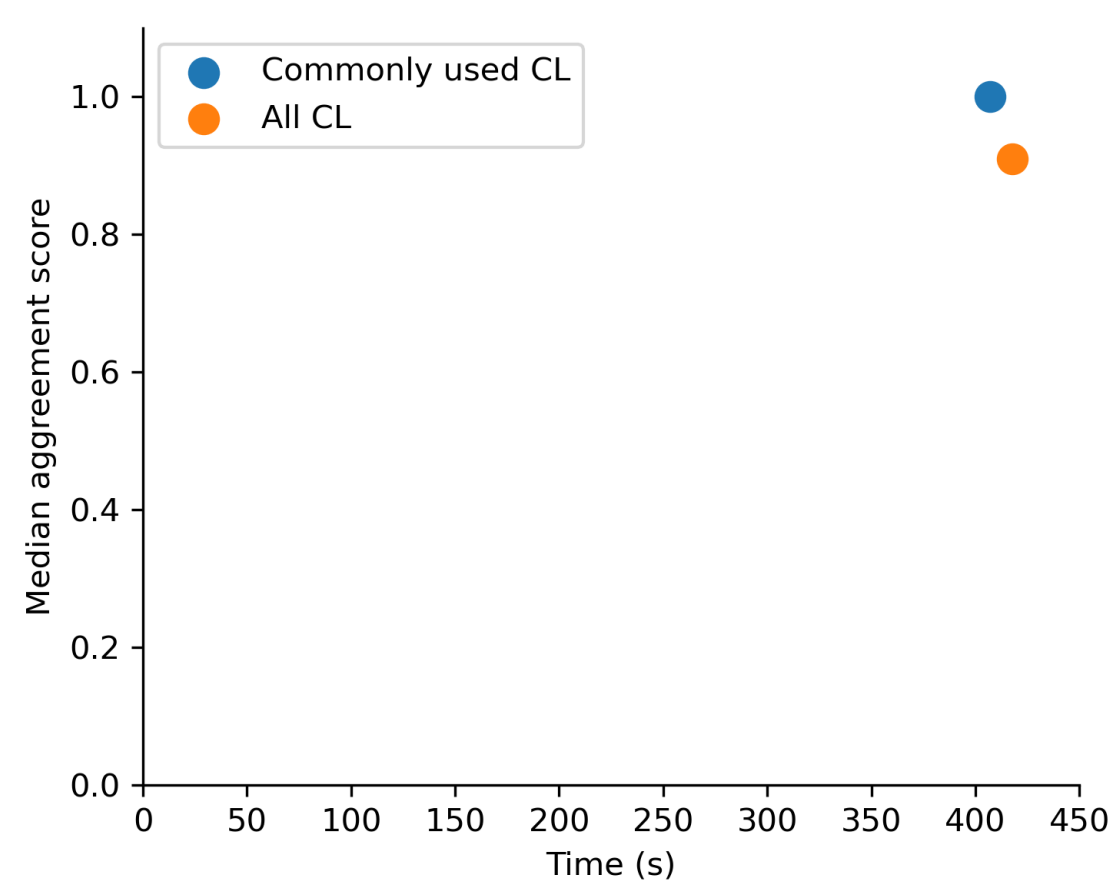

Figure S3. Median agreement score and runtime comparing the use of commonly used CL terms with all CL terms in GCTHarmony two-step strategy.

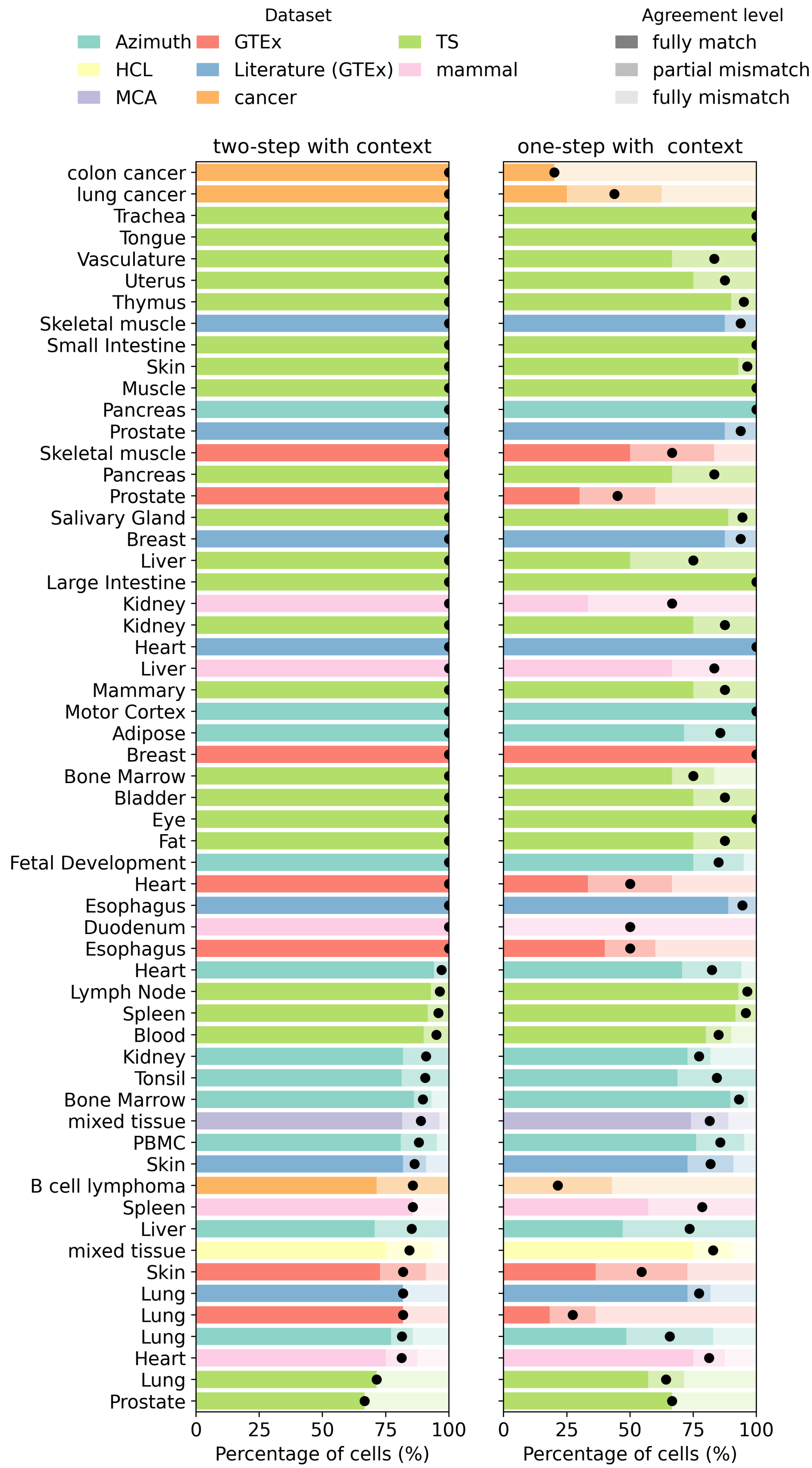

Figure S4. Proportion of cell types with different agreement levels across studies and tissues for the two-step and one-step strategies of GCTHarmony augmented by publication titles and abstracts.
